# Supplementary material for: Venomix: a simple bioinformatic pipeline for identifying and characterizing toxin gene candidates from transcriptomic data
Source: PeerJ. 2018 Jul 31;6:e5361. doi: 10.7717/peerj.5361 (PMC6074769; doi:10.7717/peerj.5361)
Supplement: Supplemental Information 3 [file peerj-06-5361-s003.gz › FinalOutput_E-6/Zinc_metalloproteinase@disintegrin_5/finaltree.pdf]

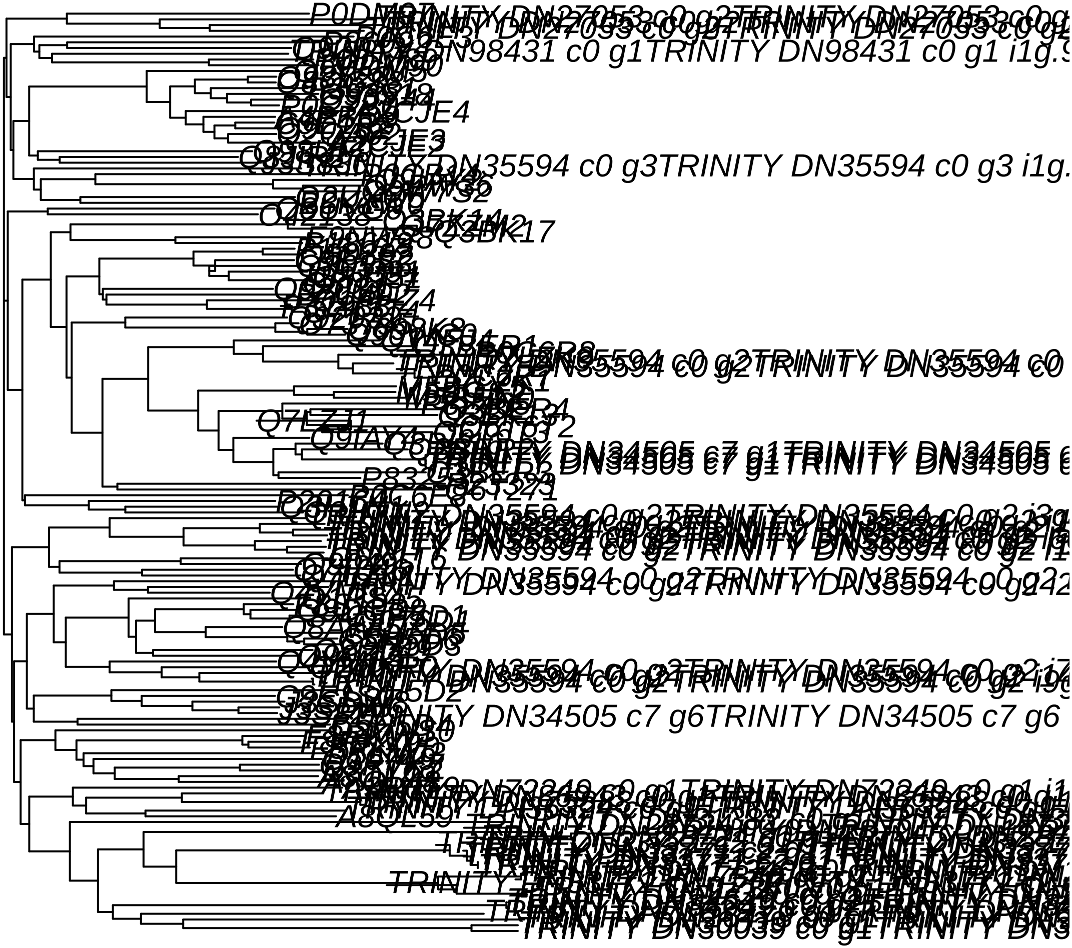
TRINITY DN34505 c7 g6TRINITY DN34505 c7 g6
Q7LZJ1
A8QL59
Q3BK17
